# Identification of gene-gene interaction using principal components

**DOI:** 10.1186/1753-6561-3-s7-s78

**Published:** 2009-12-15

**Authors:** Jia Li, Rui Tang, Joanna M Biernacka, Mariza de Andrade

**Affiliations:** 1 Department of Health Sciences Research, Mayo Clinic, 200 First Street SW, Harwick 776, Rochester, Minnesota 55905, USA

## Abstract

After more than 200 genome-wide association studies, there have been some successful identifications of a single novel locus. Thus, the identification of single-nucleotide polymorphisms (SNP) with interaction effects is of interest. Using the Genetic Analysis Workshop 16 data from the North American Rheumatoid Arthritis Consortium, we propose an approach to screen for SNP-SNP interaction using a two-stage method and an approach for detecting gene-gene interactions using principal components. We selected a set of 17 rheumatoid arthritis candidate genes to assess both approaches. Our approach using principal components holds promise in detecting gene-gene interactions. However, further study is needed to evaluate the power and the feasibility for a whole genome-wide association analysis using the principal components approach.

## Background

It is common in candidate-gene or genome-wide association studies to perform single-gene association analysis. However, after more than 200 genome-wide association studies (GWAS), there have been fewer novel loci identified than expected [1], possibly due to small effects of individual genetic variations. By supplementing GWAS data with information from previous candidate-gene or functional studies, and considering genetic interaction effects, we may be able to identify groups of genes that contribute to a complex disease. Approaches for studying gene-environment and gene-gene interactions have been proposed for the analysis of candidate genes [2,3] and genome-wide data [4]. We extend two approaches proposed for single-gene and gene-environment interaction analyses, a principal component (PC) approach [5] and a two-step approach [6], to gene-gene interaction analysis. We compare these two approaches with the traditional approach of testing all pairwise single-nucleotide polymorphism (SNP) interactions to assess gene-gene interaction effects on rheumatoid arthritis in the North American Rheumatoid Arthritis Consortium (NARAC) data.

## Methods

### Data

All of our analyses utilized genotype data of the 868 cases and 1194 controls in the NARAC data set. Analyses were carried out on a set of 17 candidate genes, selected on the basis of a literature search. The candidate genes used in these analyses are listed in Table 1. We identified all SNPs in the gene and within 5 kb on each side of each of these genes. SNPs with call rate ≤ 95% or not in Hardy-Weinberg equilibrium (*p *< 0.001) were excluded from all analyses, leading to a final set of 135 SNPs. Before analysis, the computer program MACH [7] was used to impute missing genotypes.

**Table 1:** List of candidate genes

Gene	Chromosome
*PADI4*	1
*PTPN22*	1
*STAT4*	2
*IL1B*	2
*CTLA4*	2
*ITGAV*	2
*IL13*	5
*VEGFA*	6
*TNF*	6
*LTA*	6
*HLA-A*	6
*HLA-B*	6
*HLA-C*	6
*IL6*	7
*TRAF1*	9
*C5*	9
*MS4A1*	11

### Approaches

#### Principal components

This approach was proposed by Gauderman et al. [5] to test for association between disease and multiple SNPs in a candidate gene. We extend this approach to test for gene-gene interaction. The procedure involves the following steps. 1) Let *g*_*lk *_be the number of minor alleles at SNP *k *for *l*^th ^subject, *l *= 1, ..., *N*, *k *= 1, ..., K. 2) Calculate the correlation matrix *R*, where *R*_*ij *_= *cor*(*g*_*i*_, *g*_*j*_) and *g*_*i *_and *g*_*j *_represent the genotypes of all subjects for SNP *i *and SNP *j*, respectively. 3) Decompose *R *by singular value decomposition: *R *= *A*Λ*A*^*T *^4) Determine the factor loading by . 5) Determine the PCs by PC = *GA*, where *G *is the standardized *N *× *K *matrix of genotypes. The standardized genotypes are calculated as: , where  is the mean genotype across subjects and  is the standard deviation.

Then, we use PCs that explain at least 80% of the variation as the gene representation to perform a gene-gene interaction analysis, by applying logistic regression to test for interaction between every combination of two PCs. Once significant PC interactions are identified, PC loadings may be used to determine the influence of a specific SNP on the PCs because the loading represents the correlation of a SNP with a component. For better visualization of the gene-PCs and their SNPs position with the LD block plots, we created a graphical display using our own function in the statistical package *R *and the computer program Haploview [8].

#### Two-step analysis

Murcray et al. [6] proposed a two-step approach for selecting SNPs involved in significant gene-environment interactions, where Step 1 consisted of a modified version of the case-only analysis [9,10], and in Step 2, the significant SNP-environment interactions identified in Step 1 were tested using logistic regression. We modified their method to detect gene-gene interactions as follows:

##### Step 1

For each pair of SNPs, we perform a test of association between the two SNPs (*g*_1_, *g*_2_) based on the approximate method to screen for epistasis implemented in PLINK [11] by combining cases and controls and coding *g*_1 _and *g*_2 _as 0, 1, or 2, representing the number of minor alleles. A *χ*^2 ^with 1 degree of freedom is used to test the association between each pair of SNPs. Pairs of SNPs are selected for analysis in Step 2 if they exceed a given significance threshold, *p *<α*. In our case, we selected α* = 0.05.

##### Step 2

The *M *significant SNP pairs from Step 1 are tested in a traditional log-additive model with gene-gene interaction

where *D *represents the cases (*D *= 1) and controls (*D *= 0). An interaction is considered significant when the *p*-value of interaction (i.e., the *p*-value for testing *H*_0_: *β*_3 _= 0) is less than or equal to *α*/*M*, where *α *= 0.05.

## Results

**Figure 1:**
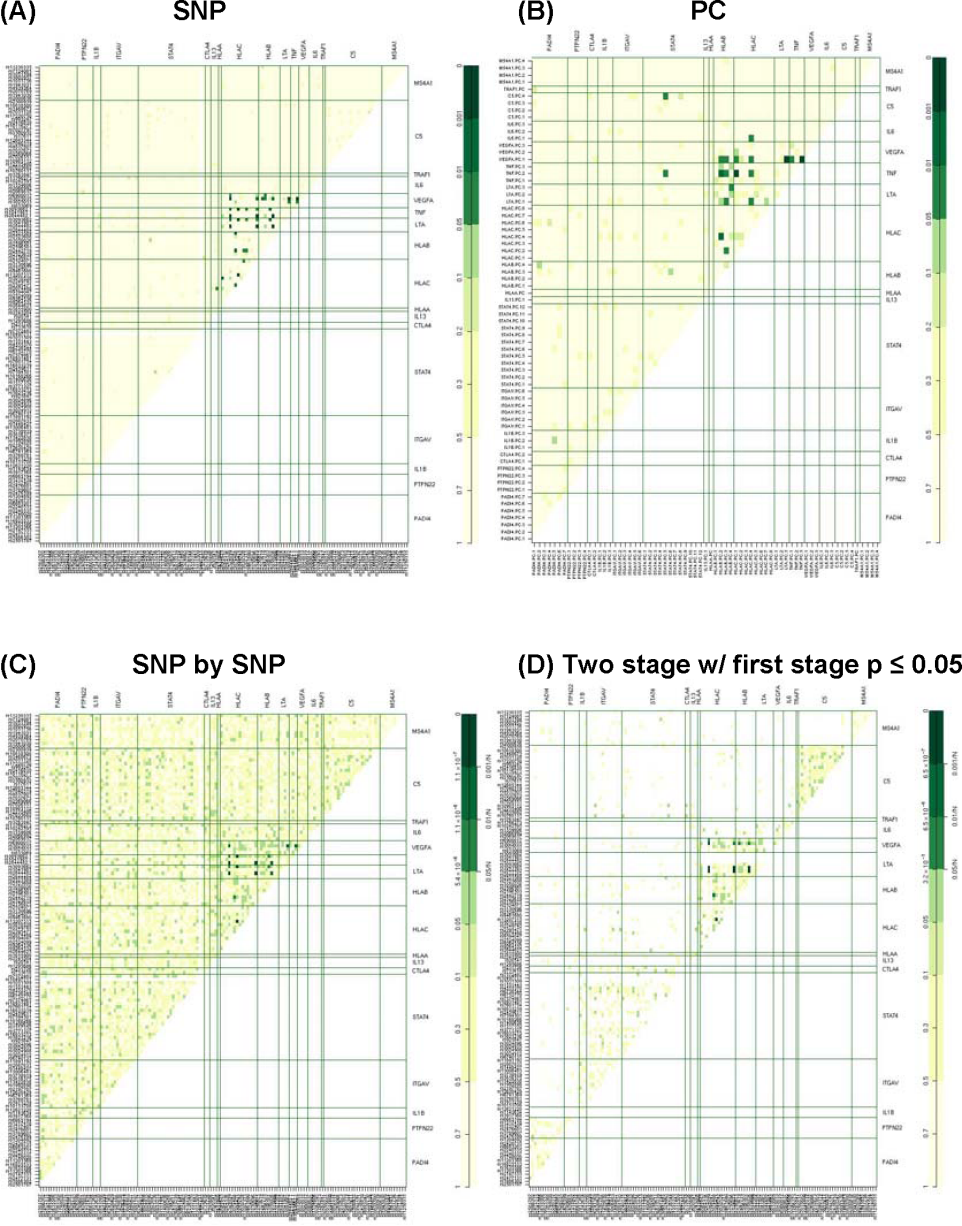
**Comparison between the gene-gene interaction approaches**. A, *Q*-values for all SNP-SNP interactions; B, PC interactions; C, *p*-values for all SNP-SNP interactions; and D, two-step approach. Darker shades of green represent smaller *p*-values.

Figure 1A shows results of all SNP-SNP interactions compared with the PC-PC interaction approach (Figure 1B) for each gene using *Q *values [12]. Figure 1C depicts the results of all SNP-SNP interactions for each gene using Bonferroni-corrected *p*-value < 5.5 × 10^-6 ^(the *α *value of 0.05 divided by *K*(*K*-1)/2 with *K *= 135 SNPs) compared with the two-stage approach with *p*-value in the first stage < 0.05 and a *p*-value in the second stage < 3.2 × 10^-5 ^(the *α *value of 0.05 divided by *M *= 1655 significant SNP pairs from Step 1).

**Figure 2:**
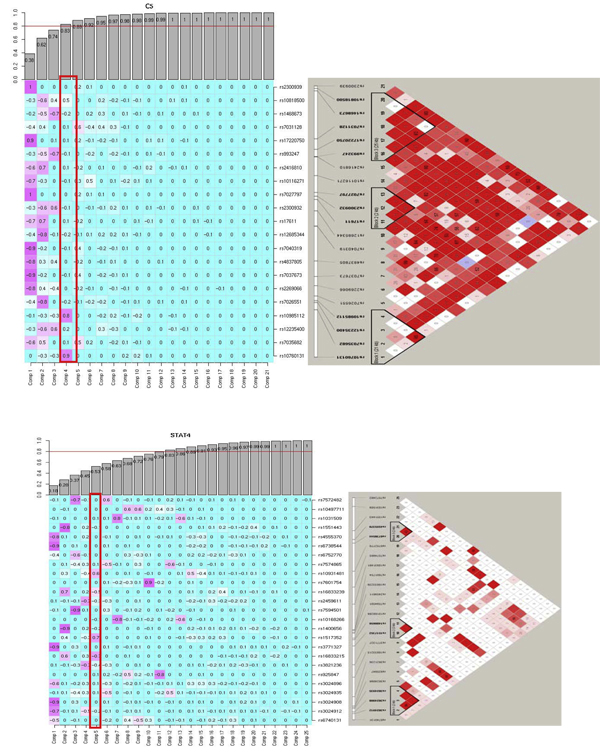
**Visualization from PC to Haploview**. Figures on the left depicted the *C5 *and *STAT4 *genes PCs with their respective loadings. The bar plot represents the cumulative percentage of explained data variance for each PC. The red blocks are the interactions of *C5-PC4 *and *STAT4-PC5*. Figures on the right depicted the *C5 *and *STAT4 *Haploview display.

The PC approach detected several PC interaction effects when using *Q*-value only. The strongest interactions were observed within the *HLA *region with *TNF-PC3 *and *VEGFA-PC1 *and *HLA-C-PC1 *and *TNF-PC2 *(*q*-value < 0.001). Outside the *HLA *region we observed two moderate interaction effects involving *STAT4-PC5 *and *C5-PC4*, and *TNF-PC2 *and *STAT4-PC5 *(0.001 <*q*-value < 0.01). Figure 2 depicts the SNP factor loadings for each PC within the genes *STAT4 *and *C5*, and the linkage disequilibrium (LD) blocks within these genes. The *STAT4-PC5 *interaction contains four SNPs with absolute value of loadings ≥ 0.5 and they represent their own block. The *C5-PC4 *interaction contains three SNPs with loadings ≥ 0.5, where the two SNPs (rs10760131 and rs10985112) with the two highest loadings, 0.9 and 0.8 respectively, belong to the same block. On the other hand, the two-step approach detected only interactions within the *HLA *region. The strongest interactions were between SNPs of *VEGFA *with *HLA-C*, *LTA *with *HLA-B*, and with *HLA-C*, and *HLA-B*, with *HLA-C*. Code to perform these analyses is available from the authors by request.

## Discussion

We extended two approaches previously used for gene-level tests or gene-environment interaction analysis to screen for gene-gene interactions in 17 candidate genes for RA using the GAW16 NARAC data. In the PC approach we calculated the SNP loadings for each PC and viewed them in the context of the gene LD structure generated using Haploview (Figure 2). This comparison is useful to identify the contribution of each SNP in the PCs and its position in the gene. For the PC gene-gene interaction analysis we used PCs that explained 80% of the variation to limit the number of PCs. Using this method we identified several gene-gene interactions. Further study to investigate the power of this PC approach is needed. This approach has potential to be used as a screening tool to detect gene-gene interaction. Subsequently, a more detailed interaction analysis should be performed using the SNPs with higher loadings [13].

We could not identify any significant interactions using the two-step approach. There are several possibilities, including the elimination of SNPs with low allele frequency, and the choice of α* in Stage 1. Recently, a similar two-step method was proposed and shown to be more powerful than a one-step approach [14]. Further evaluation of this approach is warranted.

## Conclusion

Using PCs is a promising approach to screen for potential interactions. As shown in our results, it can detect interactions not observed based on SNP-SNP interactions assessed using either a single-step or a two-step approach. Furthermore, the method used to correct for multiple comparison also plays an important role.

## List of abbreviations used

GAW16: Genetic Analysis Workshop 16; GWAS: Genome-wide association studies; LD: Linkage disequilibrium; NARAC: North American Rheumatoid Arthritis Consortium; PC: Principal components; SNP: Single-nucleotide polymorphism

## Competing interests

The authors declare that they have no competing interests.

## Authors' contributions

JL carried out the candidate genes selection, programming, and performed the statistical analysis. RT participated in the candidate genes selection and programming. JMB participated in the design of the study and helped to draft the manuscript. MdA conceived of the study, participated in its design and coordination, and drafted the manuscript. All authors read and approved the final manuscript.
